# Cooperative Oligomeric Peptide Combinations Enhance the Predicted Therapeutic Profile of SET-M33

**DOI:** 10.3390/antibiotics15060591

**Published:** 2026-06-09

**Authors:** Ismael Castanon, Giovanni Cappello, Alessandro Rencinai, Laura Cresti, Eva Tollapi, Chiara Falciani, Alessandro Pini

**Affiliations:** 1 Department of Chemistry, Bioscience and Environmental Engineering, University of Stavanger, 4014 Stavanger, Norway; 2 Department of Medical Biotechnology, University of Siena, 53100 Siena, Italy; giovanni.cappello2@unisi.it (G.C.); alessandro.rencin@student.unisi.it (A.R.); laura.cresti2@unisi.it (L.C.); eva.tollapi@student.unisi.it (E.T.); chiara.falciani@unisi.it (C.F.); pinia@unisi.it (A.P.); 3 SetLance SRL, Via Fiorentina 1, 53100 Siena, Italy; 4 Clinical Pathology Unit, Azienda Ospedaliera, Universitaria Senese, 53100 Siena, Italy

**Keywords:** antimicrobial peptides, dendrimeric peptides, peptides pool, rational design, molecular dynamics

## Abstract

**Background/Objectives**: Antimicrobial peptides (AMPs) are promising candidates against multidrug-resistant bacteria, although their clinical translation is frequently limited by cytotoxicity. In this study, we investigated whether combinations of structurally related oligomeric analogs could cooperatively enhance bacterial membrane targeting while redistributing the associated cytotoxic burden. **Methods**: Monomeric, dimeric, and tetrameric AMPs were evaluated through antimicrobial susceptibility testing, checkerboard interaction assays, RAW 264.7 macrophage cytotoxicity assays, and all-atom molecular dynamics simulations, including biased membrane insertion and umbrella sampling analyses. In addition, we introduced the Combinatorial Therapeutic Index (CTI) as an exploratory metric to estimate the predicted reduction in cytotoxic burden associated with peptide combinations. **Results**: Cytotoxicity varied substantially among oligomeric forms, with larger and more hydrophobic peptides, particularly tetramers, exhibiting the highest cytotoxicity. Additive effects were observed in checkerboard assays involving linear, dimeric, and tetrameric forms, supporting the redistribution of the toxic burden and enabling the beneficial membrane-interaction properties of hydrophobic linear peptides to be leveraged at lower cytotoxic cost. Predicted therapeutic improvement ranged from approximately twofold for the SET-M33:L33 combination to nearly ninefold for the SET-M33:DIM-33:L8 triple combination. Molecular dynamics simulations revealed non-redundant membrane interaction behaviors, with smaller peptides exhibiting deeper membrane insertion and the dimeric form favoring interfacial membrane engagement. **Conclusions**: These findings support a cooperative formulation strategy in which structurally related SET-M33 oligomers contribute complementary antibacterial functions while reducing the predicted cytotoxic burden. Further experimental validation using direct cytotoxicity assays of complete peptide mixtures will be necessary to confirm the therapeutic potential of these formulations.

## 1. Introduction

Antimicrobial peptides (AMPs) are characterized by their often-restricted activity spectrum, which derives from a generalist mechanism of action against the bacterial envelope [1,2,3,4]. Unlike many small molecules, which usually target defined enzymes or intracellular pathways, antimicrobial peptides frequently interact with larger and more heterogeneous structures, such as the bacterial membrane and cell envelope. This mode of action is attractive because it targets essential and broadly conserved features of bacterial physiology, potentially reducing the probability of resistance development. However, the same property also complicates the development of broad-spectrum clinical candidates, since bacterial envelopes differ substantially among species and even among strains.

High cytotoxicity, together with poor stability, has represented one of the main difficulties behind AMP clinical translation, and it has relegated many candidates to limited or specialized therapeutic applications, restricting the possible administration pathways [5,6,7]. Biomaterials, external medical devices, topical formulations, and intravenous administration are some of the common fields in which AMP research is being explored; however, even in these contexts, toxicity remains one of the main concerns [8,9,10,11,12,13].

Innovative strategies are being developed to tackle some of the aforementioned problems. D-amino acids have been demonstrated to be less susceptible to proteases, especially when they are included in the core of the peptide sequence [14,15]. Oligomerization and cyclization have also been widely documented in the literature and have effectively increased the stability of L33 when tetramerized into SET-M33, the model peptide of this study [16,17,18,19,20]. PEGylation and nanoparticle encapsulation have also helped with AMP administration, improving delivery, circulation time, or local accumulation depending on the formulation [21,22]. However, despite these advances, the main obstacles behind their clinical development are still present, particularly the difficulty of improving antimicrobial potency without simultaneously increasing toxicity or reducing specificity.

This limitation largely derives from the fact that effective antimicrobial activity against different bacterial membranes requires partially conflicting physicochemical properties. Gram-negative bacteria differ substantially in lipid A organization, phospholipid composition, membrane charge density, and lipid packing, all of which can strongly influence peptide binding, insertion, and membrane disruption efficiency [23,24,25,26,27,28]. Consequently, the physicochemical profile that maximizes activity against one membrane environment may not be optimal for another.

Traditional AMP optimization strategies attempt to balance these constraints through rational sequence modification, typically by adjusting cationicity, hydrophobicity, helicity, or amphipathicity. However, these properties are often interdependent and functionally opposed. For example, increasing hydrophobicity or helicity may improve membrane insertion and antimicrobial potency, but frequently at the cost of increased hemolysis or mammalian cytotoxicity. Similarly, increasing positive charge can strengthen electrostatic membrane association, while excessive cationicity may compromise selectivity or alter pharmacokinetic behavior [29,30,31,32,33,34]. As a result, optimization of a single peptide architecture may eventually reach a functional trade-off limit in which further improvement in one property leads to deterioration in another.

Cooperative antimicrobial strategies have previously been described in AMP-antibiotic combinations, synergistic membrane-permeabilizing peptides, and naturally occurring two-component bacteriocin systems, where distinct molecules contribute complementary antimicrobial functions [35,36,37]. However, these approaches generally rely on mechanistically differentiated compounds rather than structurally related oligomeric variants intentionally designed to redistribute physicochemical constraints and cytotoxic burden across complementary peptide architectures.

Based on this rationale, we hypothesized that these functions could instead be distributed across structurally related peptide variants with complementary properties. Under this framework, the antimicrobial effect would not depend exclusively on a single optimized molecule, but rather on the cooperative contribution of different peptide species. In such combinations, individual components may contribute distinct roles, while their proportions can be adjusted to preserve antibacterial activity and reduce the toxic burden associated with the most cytotoxic variants. We tested this concept using oligomeric variants of SET-M33, asking whether cooperative mixtures could improve the predicted therapeutic profile by redistributing antimicrobial load away from the most cytotoxic species.

## 2. Results

### 2.1. Structural and Functional Impact of Cationic Scaling and Hydrophobic Disruption in SET-M33 Analogs

The well-characterized tetrameric antimicrobial peptide SET-M33 was utilized as a scaffold for a lysine/arginine scanning experiment, and its analogs were systematically evaluated against a carefully selected panel of clinically relevant bacterial strains, including clinically relevant Gram-negative bacteria such as *P. aeruginosa*, *K. pneumoniae*, and *E. coli* [38,39,40,41].

**Figure 1:**
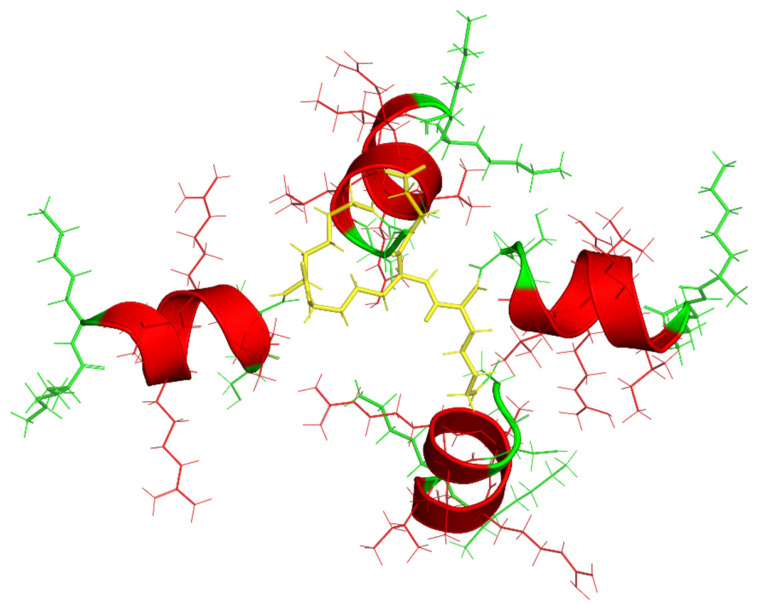
Representative structural model of the tetrameric antimicrobial peptide SET-M33, highlighting key motifs involved in activity. The α-helical region formed by residues 3–7 (“IRVRL”) is shown in red, while the poly-lysine branching core is shown in yellow. The structure was obtained from all-atom molecular dynamics simulations performed in GROMACS and is shown for structural visualization purposes.

All evaluated sequences corresponded to structural analogs of the tetrabranched peptide SET-M33, which consists of four identical peptide chains covalently linked to a poly-lysine core (Figure 1). In the present work, a cationic scanning strategy was implemented to explore two complementary hypotheses: first, to assess the potential benefit of increasing the net positive charge in enhancing electrostatic interactions with the negatively charged bacterial membranes, and second, to evaluate how specific hydrophobic disruptions along the peptide chain could affect the structural integrity and bioactivity of the molecule. This experiment provided information about the physicochemical profile of SET-M33 for subsequent optimization.

The initial screening revealed significant variations in antimicrobial potency among the different peptide analogs, with MIC values ranging from sub-micromolar to ineffective concentrations depending on both the peptide sequence and target bacterial species (Table 1). Substitutions within the central IRVRL motif were associated with dramatically reduced activity, exhibiting MIC values of 50 μg/mL or higher (see Figure 1). Outside this motif, both terminal substitutions retained most of their activity. Notably, the RKIRVRLSA sequence preserved strong activity against *Pseudomonas aeruginosa* PAO-1 and *Escherichia coli* TG-1 (MIC = 1.5 μg/mL for both), showing comparable efficacy to SET-M33 (KKIRVRLSA) in these strains, but exhibited reduced potency against *Klebsiella pneumoniae* ATCC (MIC = 6 μg/mL for RKIRVRLSA). Similar effects were observed for analogs KKIRVRLKA and KKIRVRLRA, which, near C-terminal substitutions, retained most of their efficacy against *Pseudomonas aeruginosa* PAO-1 and *Escherichia coli* TG-1 (MIC = 3 μg/mL for both), while presenting reduced potency against *Klebsiella pneumoniae* ATCC (MIC = 6 μg/mL for both). The final two analogs, KKIRVRLSK and KKIRVRLSR, exhibited an interesting activity profile. KKIRVRLSK displayed the lowest MIC recorded against *P. aeruginosa* PAO-1 across the entire panel (0.70 μg/mL), surpassing even the parental SET-M33, while retaining moderate activity against *E. coli* TG-1 (3.00 μg/mL). However, this gain in potency against Gram-negative non-enteric pathogens came at a cost: activity against *K. pneumoniae* ATCC collapsed to 50.00 μg/mL. Interestingly, KKIRVRLSR showed a general reduction in activity across all tested strains, with MIC values of 25.00 and 12.00 μg/mL against *P. aeruginosa* and *E. coli*, respectively, and no measurable activity against *K. pneumoniae*, indicating that arginine at this terminal position is poorly tolerated regardless of the target species.

### 2.2. Structure-Preserving Sequence Optimization of SET-M33 Analogs to Probe Peripheral Physicochemical Determinants of Activity

As previously established, understanding the functional requirements of antimicrobial peptides depends on accurately characterizing peptide-membrane interactions. These interactions involve several simultaneous processes, including initial electrostatic binding, membrane insertion, conformational flexibility, and ultimate membrane permeabilization, each of which is linked to the physicochemical profile of the peptides.

To characterize the current profile of SET-M33 and identify opportunities for further optimization, we implemented an exploratory rational design strategy, which aimed to analyze how peripheral substitutions modulate the activity of SET-M33. To do this, we designed six different tetrameric SET-M33 analogs utilizing CAMP-R4 machine learning platforms for their design [42]. Three analogs maintained the same net positive charge as SET-M33 but displayed increased aliphatic index, while the other three analogs preserved the original hydrophobicity but incorporated additional cationic residues to increase the overall net charge. This design enabled the evaluation of structure-activity relationships (SARs) by modulating electrostatic and hydrophobic interactions independently from the helical core.

The results showed that analogs with enhanced cationic charge density exhibited greater retention of antimicrobial activity across all tested Gram-negative pathogens (*E. coli*, *P. aeruginosa*, and *K. pneumoniae*), reflecting the fact that peripheral net charges were relevant for their action mechanism.

Notably, bacterial-specific responses were observed, with *K. pneumoniae* showing a more pronounced decrease in sensitivity compared to *E. coli* and *P. aeruginosa*. This effect was likely related to differences in membrane composition, particularly the lower abundance of anionic phospholipids such as cardiolipin in *K. pneumoniae,* which limited the efficiency of electrostatic interaction and membrane disruption by cationic peptides.

Among the synthesized analogs, KKIRVRLKLK retained substantial antimicrobial activity, exhibiting an MIC value of 3 μg/mL against *K. pneumoniae* and 1.5 μg/mL against *E. coli*; these findings indicate that selective peripheral modifications could improve efficacy when the structural scaffold is respected. In contrast, the KKIRVRLSI analog, designed with a hydrophobic substitution outside the motif but without increased charge, showed marked loss of activity across all strains (Table 2). This suggested that even non-core modifications could negatively impact overall peptide behavior, potentially through subtle effects on amphipathicity, solubility, or peptide-membrane orientation.

More broadly, the relationship between net charge and antimicrobial activity was not strictly proportional across all evaluated strains, suggesting that antimicrobial efficacy may also depend on the spatial distribution of cationic residues and peptide structural context.

### 2.3. Cytotoxicity Analysis and Structure-Activity Relationships

Hydrophobicity has been widely investigated in the literature as an important determinant for linear antimicrobial peptides, but to further define the cytotoxic boundaries of the rational design of dendrimeric peptides, we analyzed the cytotoxic effects of a SET-M33 analog library on eukaryotic cells using the RAW 264.7 macrophage cell line, which serves as a well-established model for assessing peptide toxicity in mammalian systems. Although mammalian and bacterial membranes differ substantially in composition, membrane-active physicochemical determinants such as hydrophobicity and amphipathicity are known to strongly influence peptide interactions in both systems [43].

The batch of analogs analyzed comprises 6 peptides that exhibit an aliphatic index range from 97 to 162. This index describes the contribution of aliphatic residues (Ala, Val, Ile, and Leu) to the peptide’s overall composition and is demonstrated to be closely linked to mammalian toxicity.

**Figure 2:**
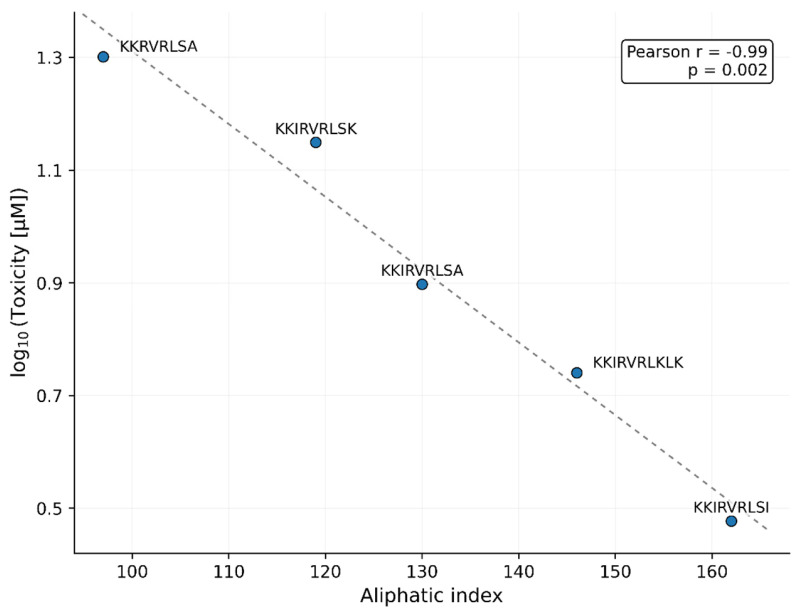
Correlation between toxicity-related concentration values (EC50) and aliphatic index among structurally related SET-M33 analogs evaluated in RAW 264.7 murine macrophages. Analog 1 (KKRVRLSA) lacks the N-terminal Ile residue, Analog 2 (KKIRVRLSK) contains a C-terminal Lys substitution, Analog 3 corresponds to the parental SET-M33 sequence (KKIRVRLSA), Analog 4 (KKIRVRLKLK) includes a Lys-Leu-Lys extension, and Analog 5 (KKIRVRLSI) contains a C-terminal Ile substitution. Toxicity values were obtained from MTT-based dose-response viability assays.

Across the five analogs, the aliphatic index showed a strong inverse association with the toxicity-related readout (Pearson r = −0.99, *p* = 0.002; Figure 2), supporting a clear structure-toxicity relationship within this series. Using the original peptide KKIRVRLSA (Analog 3; SET-M33; aliphatic index = 130) as the reference sequence, Analog 1 (KKRVRLSA; aliphatic index = 97) lacks the N-terminal Ile, Analog 2 (KKIRVRLS*K*; aliphatic index = 119) retains the same core scaffold but replaces the C-terminal Ala with Lys, Analog 4 (KKIRVRL*KLK*; aliphatic index = 146) introduces a C-terminal Lys-Leu-Lys extension, and Analog 5 (KKIRVRLS*I*; aliphatic index = 162) replaces the C-terminal Ala with Ile. Together, these data indicated that even limited sequence changes relative to the original KKIRVRLSA scaffold could markedly alter the aliphatic characteristic of the peptide and its associated mammalian-cell response.

**Figure 3:**
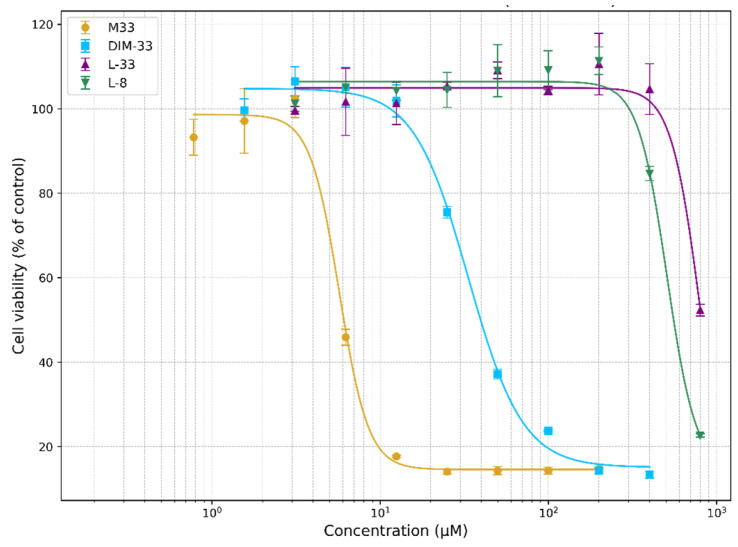
Cytotoxic activity of SET-M33 oligomers against RAW 264.7 murine macrophages. Cell viability (% of untreated control) was assessed across a concentration range of 0.5-800 µM for the tetrameric (SET-M33), dimeric (DIM-33), and monomeric (L-33, L-8) forms. Data points represent mean ± SD of three independent experiments. Solid lines correspond to four-parameter sigmoidal Hill fits. CC50 values were 5.7 µM (SET-M33), 33.5 µM (DIM-33), 508 µM (L-8), and 801 µM (L-33).

The second batch of analogs comprised four peptide oligomers (SET-M33, DIM-33, L33, and L8), which were also evaluated against RAW 264.7 murine macrophages using a dose-response viability assay via MTT colorimetric assay (Figure 3). All compounds followed a sigmoidal Hill-type inhibition curve, although exhibiting different potencies depending on the oligomerization state. Although a fully defined upper viability plateau was not observed for the least cytotoxic peptides within the evaluated concentration range, the obtained dose-response profiles were sufficient to establish robust comparative EC50 trends among the evaluated oligomeric forms.

SET-M33 was determined as the most cytotoxic form, exhibiting a CC50 of 5.7 µM, while its dimeric form reduced its toxicity to 33.5 µM. The linear peptides L8 and L33 displayed substantially reduced cytotoxicity, with CC50 values of 508 and 801 µM, respectively, exhibiting essentially non-toxic profiles across the entire pharmacologically relevant concentration range.

### 2.4. Fractional Inhibitory Concentration Analysis and Synergistic Interactions

Antimicrobial efficacy testing revealed distinct patterns between peptide structure, hydrophobicity, and individual MIC values against *E. coli* TG1. L33 (KKIRVRLSA), the linear version of SET-M33, as well as its most hydrophobic analog, L8, which exhibited a MIC value of 3 μM. The dimeric form of L33, DIM-33, showed higher efficacy (0.75 μM MIC), and the tetrameric counterparts SET-M33 and SET-M8 showed distinct MIC efficacy of 0.35 μM and 3 μM, respectively, exemplifying how the oligomeric structure compromised the sequence influence on activity, modifying the functional bottlenecks of peptides (Table 3).

**Figure 4:**
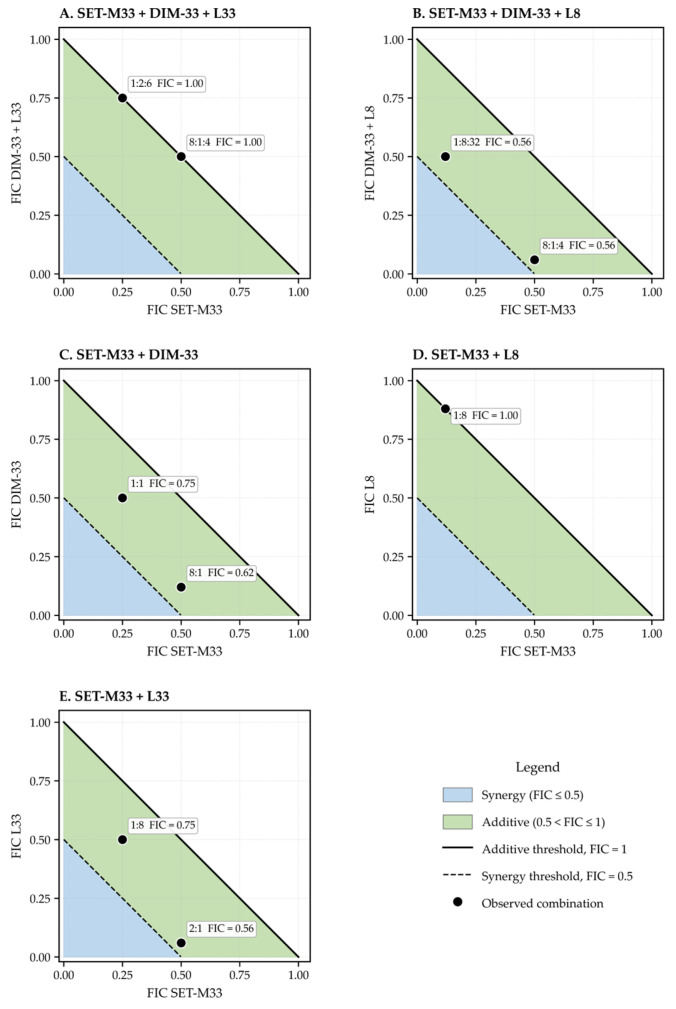
Isobolograms showing fractional inhibitory concentration (FIC) interactions among tetrameric SET-M33 and structurally related oligomeric analogs. Dashed and solid lines indicate the theoretical synergy (FIC = 0.5) and additive (FIC = 1) thresholds, respectively. Blue and green regions represent synergistic and additive interactions. Experimental combinations are shown as black circles.

Checkerboard assays of tetrameric SET-M33 and the monomeric form L33 revealed distinct interaction patterns. The combination of tetrameric SET-M33 with its linear monomeric form demonstrated strong additive effects that decreased as the ratio shifted toward higher monomer concentrations. At optimal ratios favoring the tetrameric component, the combination achieved FIC = 0.56, indicating robust additive behavior approaching the synergy threshold. However, as the monomer proportion increased, the FIC value deteriorated to 0.75, showing reduced but still additive combinatorial efficacy (Figure 4).

The combination of SET-M33 and L8 exhibited markedly different behavior, showing only slight additive effects. The 1:8 ratio achieved FIC = 1.0, representing weak additive behavior at the boundary between additive and indifferent classification. This additive interaction weakened as the L8 concentration decreased, indicating that the linear form needed to be at a substantial concentration within the combination to present some additive antibacterial contribution.

The combination of DIM-33 with its tetrameric form, SET-M33, produced a similar effect to that previously observed with the linear monomeric counterpart. However, the dimeric form required significantly lower concentrations relative to the monomer to achieve equivalent FIC values. At a 1:1 ratio, the tetramer-dimer combination yielded FIC = 0.73, while a 1:0.1 ratio achieved FIC = 0.60, demonstrating that optimal dimer performance occurred at concentrations approximately 8-fold lower than required for equivalent monomeric efficacy.

This concentration difference reflects structural conformational limitations imposed by the larger size of dimeric forms, which may experience spatial constraints during membrane organization compared to the more flexible monomeric structures.

Triple combinations incorporating tetrameric, dimeric, and monomeric components were systematically evaluated to assess multi-component interactions and their potential for therapeutic optimization.

When the combination was based on the use of SET-M33, DIM-33 and L33, the combination exhibited weak additive behavior (FIC = 1.0). This behavior was consistently observed at every pool concentration tested, indicating that the additive effect observed in dual combinations became attenuated when both dimeric and monomeric forms were simultaneously included.

Surprisingly, when the most hydrophobic linear peptide L8 was combined with SET-M33 and DIM-33, a different behavior was observed. Low concentrations of the monomeric and dimeric forms retained additive antibacterial activity (FIC = 0.56), close to the synergy threshold (FIC = 0.56), suggesting that the L8-containing triple combination benefited from a physicochemical balance that was not observed during dual combination. Moreover, this additive effect is maintained as the tetramer concentration decreases, indicating that the pool achieves the same antibacterial endpoint with a lower contribution from SET-M33, the most cytotoxic oligomer in the series.

### 2.5. All-Atom Molecular Dynamics Analysis of Antimicrobial Peptide Insertion

All-atom molecular dynamics simulations were used to compare the membrane interaction behavior of L33, L8, and DIM-33 under a standardized biased-insertion protocol. SET-M33 was simulated under the same conditions but was excluded from the main quantitative insertion analysis because systematic periodic-image interactions were detected due to its larger molecular size relative to the simulation box. No relevant trajectory discontinuities, peptide fragmentation, membrane tearing, or abnormal box instabilities were observed for L33, L8, or DIM-33; detailed quality-control analyses are reported in the Supplementary Information.

**Figure 5:**
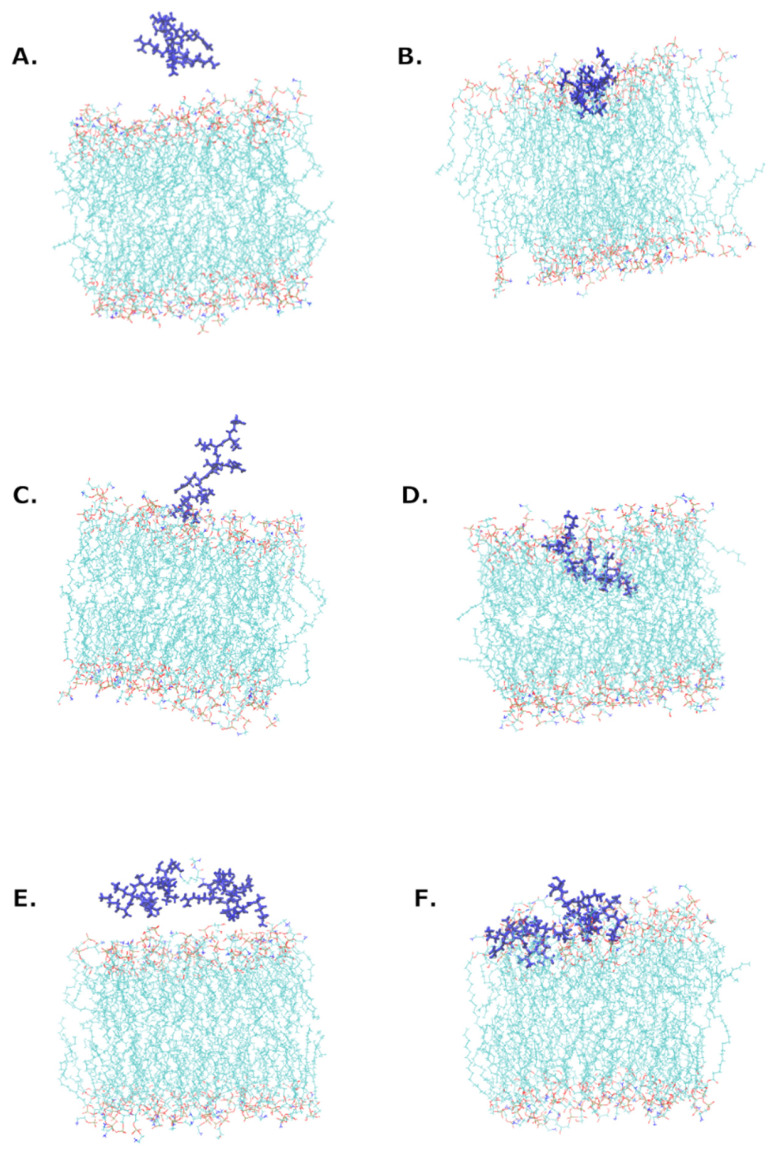
Snapshots prior to and after biased insertion for all-atom simulation in GROMACS for L33, L8 and DIM-33. Panel (**A**,**B**) refers to L33, panel (**C**,**D**) refers to L8, and panel (**E**,**F**) refers to DIM-33. The membrane is depicted in cyan (lipid tails) and red (headgroups), and peptides are shown in blue.

Representative structures before and after membrane insertion showed clear peptide-dependent differences in membrane engagement and conformational adaptation (Figure 5). The linear peptides reached deeper membrane-associated states than the dimeric form, whereas DIM-33 remained preferentially positioned closer to the membrane interface. These qualitative differences were consistent with the quantitative center-of-mass distance analysis.

**Figure 6:**
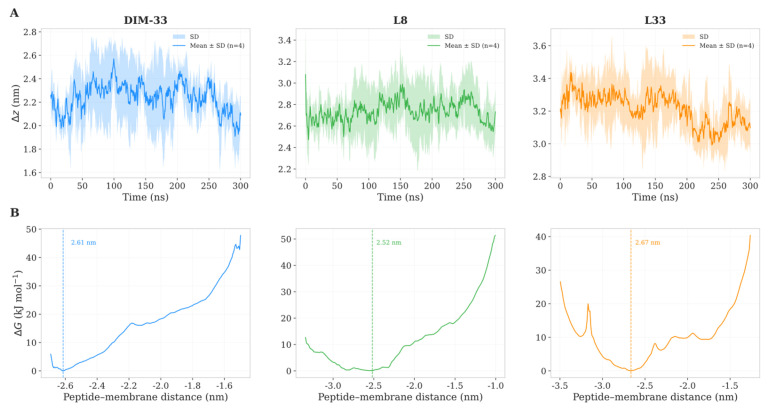
Membrane insertion analysis of antimicrobial peptides over 300 ns. (**A**) Time evolution of the center-of-mass distance (Δz) between each peptide and the membrane along the *z*-axis (mean and SD, n = 4 replicas). Lower Δz values indicate deeper membrane insertion. (**B**) Potential of mean force (PMF) profiles obtained from umbrella sampling simulations describing peptide-membrane interaction energetics as a function of peptide-membrane distance.

L8 displayed the deepest membrane insertion among the three main systems, reaching a final peptide-membrane center-of-mass distance of 1.65 ± 0.35 nm. L33 showed a slightly more superficial insertion profile, with a final distance of 1.80 ± 0.26 nm. Lastly, DIM-33 remained the least inserted peptide, with a final distance of 2.10 ± 0.22 nm (Figure 6A). Peptide orientation also differed between systems. L8 adopted a predominantly interfacial orientation with a high tilt angle relative to the membrane normal (79.6 ± 9.9), consistent with its lower helical structure, while L33 displayed a more oblique orientation (63.4 ± 4.5). Interestingly, DIM-33 showed high tilt-angle values (88.3 ± 14.0) despite remaining more superficial, suggesting that its larger oligomeric architecture constrains deeper penetration into the bilayer.

Umbrella sampling simulations supported these insertion trends by revealing distinct free-energy profiles for each peptide (Figure 6B). All three systems exhibited free-energy minima within the outer sub-membrane locations (from 2.52 to 2.67 nm). L8 showed the shallowest minimum along the reaction coordinate, at 2.52 nm, followed by DIM-33 at 2.61 nm and L33 at 2.67 nm. The L8 profile displayed a broad and smooth energy well, consistent with a favorable insertion pathway. DIM-33 showed a narrower profile, compatible with a more geometrically constrained insertion mode. L33 exhibited a more complex profile, including local barriers around 3.2 nm, corresponding to the interfacial region.

**Figure 7:**
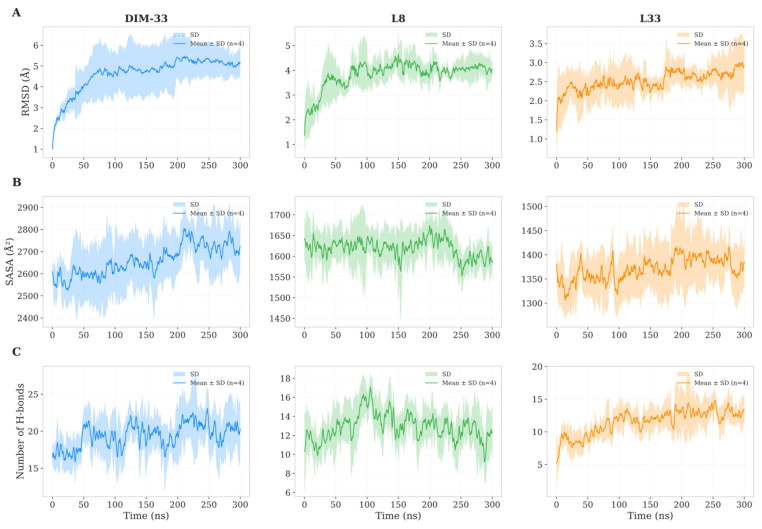
Structural dynamics of antimicrobial peptides at the bacterial membrane over 300 ns (mean ± SD, n = 4 replicas). (**A**) Root mean square deviation (RMSD) of the peptide backbone relative to the initial structure, used as an indicator of conformational flexibility. (**B**) Solvent-accessible surface area (SASA) over time, reflecting progressive membrane burial. (**C**) Number of intermolecular hydrogen bonds between each peptide and membrane phospholipids.

Structural dynamics further distinguished the peptide variants during membrane interaction (Figure 7A). DIM-33 showed the highest mean RMSD, reaching a plateau of 4.63 ± 0.92 Å after approximately 50 ns, indicating larger conformational rearrangements and higher inter-replica variability. L8 stabilized at 3.84 ± 0.25 Å, whereas L33 reached a lower RMSD plateau of 2.52 ± 0.17 Å, consistent with greater structural rigidity during membrane association. Despite its higher absolute RMSD, DIM-33 displayed slower and more correlated conformational changes, suggesting gradual adaptation rather than rapid local fluctuations.

Solvent-accessible surface area analysis revealed different burial dynamics across peptide architectures (Figure 7B). L8 and DIM-33 showed similar per-residue SASA reductions, with values of −10.6 ± 6.2 and −10.8 ± 34.6 Å^2^ per residue, respectively. L33 showed a smaller decrease of −5.2 ± 6.5 Å^2^ per residue, consistent with a premature helical structure. DIM-33 exhibited the highest variability again and showed partial SASA recovery after approximately 150 ns, suggesting a more dynamic conformational reorientation during the biased insertion.

Peptide-lipid hydrogen bond analysis showed that DIM-33 formed the highest absolute number of membrane hydrogen bonds (mean of 19.41 ± 1.40), reflecting its larger size and higher number of potential interaction sites (Figure 7C). Nevertheless, after normalization by peptide length, the linear peptides displayed a higher number of membrane interactions per residue. L8 showed the highest normalized hydrogen-bonding rate, followed by L33. These results indicate that although the dimeric form establishes more total contact, the monomeric peptides interact more efficiently with the membrane on a per-residue basis.

Lipid-contact analysis revealed preferential association with cardiolipin in several systems, although this was observed to have substantial inter-replica variability. Cardiolipin enrichment relative to POPG and POPE was 1.45 ± 0.44 and 2.13 ± 0.78 for DIM-33; 0.98 ± 0.54 and 1.20 ± 0.82 for L8; and 1.83 ± 1.10 and 1.23 ± 0.48 for L33, respectively. The lower cardiolipin enrichment observed for L8 may reflect its deeper penetration into the bilayer, where sustained interaction with surface-localized cardiolipin headgroups becomes less favorable.

Overall, these simulations indicate that smaller linear peptides, particularly the more hydrophobic L8, display greater insertion propensity and more efficient per-residue membrane contacts, whereas the dimeric form remains more interfacial and undergoes broader conformational adaptation. These differences support the presence of distinct membrane-interaction modes among the oligomeric forms, which may contribute to their complementary behavior in antimicrobial combinations.

### 2.6. Combination-Specific Predicted Therapeutic Profiles Using CTI

Checkerboard assays revealed that several SET-M33-based combinations preserved additive antibacterial activity while reducing the contribution of the most cytotoxic oligomeric component. The SET-M33:L33 dual combination showed the most pronounced additive behavior at the 2:1 ratio, with a FIC value of 0.56, close to the synergy threshold. This interaction weakened as the relative proportion of L33 increased, reaching FIC = 0.75 at higher monomeric contribution. A similar additive profile was observed for SET-M33:DIM-33 combinations, with FIC values ranging from 0.75 at the 1:1 ratio to 0.62 at the 8:1 tetramer:dimer ratio. In contrast, SET-M33:L8 dual combinations showed weaker additive behavior, reaching the additive/indifferent boundary with FIC = 1.0.

Triple combinations revealed a more marked dependence on the identity of the monomeric component. The SET-M33:DIM-33:L33 combination showed only weak additive behavior, with FIC values close to 1.0 across the tested ratios. In contrast, the SET-M33:DIM-33:L8 combination restored a stronger additive effect, reaching FIC ≈ 0.56 even when SET-M33 was present at a low concentration, as observed for the 1:8:32 ratio. This indicates that the hydrophobic monomer L8 contributed more favorably to the triple combination context than to the corresponding dual SET-M33:L8 combination.

Because FIC values only describe antibacterial interaction and do not account for the cytotoxicity of each component, we developed the Combinatorial Therapeutic Index (CTI), a novel index utilized to estimate the predicted reduction in relative cytotoxic burden achieved by each formulation. The SET-M33:L33 2:1 combination, which showed FIC = 0.56, produced a CTI of 2.05, indicating an approximately twofold predicted improvement relative to the equivalent standalone effective doses of the same components.

The triple SET-M33:DIM-33:L8 combination at a 1:8:32 ratio reached a similar antibacterial interaction score, and also had FIC ≈ 0.56, but produced a substantially higher CTI of 8.87. This greater effect is explained by the lower concentration of the most cytotoxic peptide within the pool: the tetramer, whose cytotoxicity was previously shown in this study (Table 3).

Together, these results show that combinations with comparable FIC values can have markedly different predicted cytotoxicity profiles. In particular, the SET-M33:DIM-33:L8 triple combination preserved additive antibacterial activity while producing the largest predicted reduction in relative cytotoxic burden.

This theoretical improvement emerges because the toxic response follows a sigmoidal Hill-type relationship in which cytotoxicity increases steeply once a critical concentration threshold is exceeded. Maintaining the tetramer dose below this threshold through oligomeric redistribution may therefore keep the system within a lower-toxicity region of the response curve, potentially leading to substantial predicted reductions in cytotoxic burden even under moderate FIC values.

Importantly, the CTI framework assumes approximately additive cytotoxic contributions among peptide components and should therefore be interpreted as an exploratory comparative model rather than a validated predictor of mixture toxicity. Although the evaluated peptide combinations showed additive antibacterial behavior and shared membrane disruption as a common mechanistic basis, nonlinear or emergent interactions within peptide mixtures could still alter the effective toxicity profile of combined formulations. Consequently, direct experimental validation of complete peptide mixtures remains necessary.

## 3. Discussion

The central outcome of this study is that the therapeutic potential of SET-M33-derived antimicrobial peptides may be improved not only by modifying peptide sequence or oligomeric state, but also by redistributing antibacterial activity across complementary peptide architectures. Rather than pursuing a single optimized analog with maximal potency and minimal toxicity, our results indicate that physicochemically refined combinations of tetrameric, dimeric, and monomeric forms can cooperate to overcome key bottlenecks associated with hydrophobicity and oligomerization. This suggests a potential framework for addressing a recurrent limitation in antimicrobial peptide development: the close coupling between membrane-disruptive potency and host-cell toxicity.

SET-M33 analysis revealed a relevant physicochemical profile for its optimization. A key central structural motif was identified spanning residues 3 to 7 in each branch, where cationic substitutions disrupt the hydrophobicity required to develop the necessary helicity for its mechanism of action. Terminal motifs proved to be better tolerated and provided different efficacy depending on the bacterial target. Notably, peripheral substitutions that increased cationic charge retained efficacy better than those that increased hydrophobicity. These findings suggest that SET-M33 activity depends on a constrained balance between electrostatic attraction, hydrophobic insertion, amphipathic organization, and oligomeric presentation.

The cytotoxicity experiments further illustrated this trade-off. The most hydrophobic tetrameric forms exhibited the highest cytotoxicity against RAW 264.7 macrophages, and this effect became even more pronounced when the corresponding linear peptides were oligomerized. Since hydrophobicity appeared to be more critical for some peptide architectures than for others, as observed between L8 and SET-M8, and because excessive hydrophobicity limits the clinical potential of antimicrobial peptides, it is reasonable to propose that hydrophobicity is not equally required for all peptides, and therefore, its role could be redistributed towards less cytotoxic peptide forms, such as the linear peptides.

Through checkerboard analysis, SET-M33 and different linear and dimeric analogs, including L8, L33, and DIM-33, were shown to retain their antimicrobial activity, while CTI toxicity studies demonstrated a redistribution of the toxicity burden. The SET-M33:L33 2:1 combination and the SET-M33:DIM-33:L8 1:8:32 combination both produced FIC values close to 0.56, yet their CTI values differed markedly. The dual SET-M33:L33 combination produced an approximately twofold predicted improvement, whereas the triple SET-M33:DIM-33:L8 combination produced a nearly ninefold predicted CTI improvement. This difference reflects the stronger reduction in the tetrameric SET-M33 contribution in the triple formulation.

This cooperative behavior was further investigated through all-atom biased peptide-insertion simulations performed in GROMACS. As the peptides became larger and more structurally constrained, they tended to insert less deeply into the membrane. The larger forms seemed to develop stronger surface engagement and electrostatic association, whereas smaller and more flexible peptides appeared to navigate the hydrophobic membrane core more efficiently. Umbrella sampling experiments provided useful insights into their insertion resistance. L8 exhibited a broad and smooth free-energy well, consistent with a favorable insertion pathway driven by hydrophobic interactions. In contrast, DIM-33 showed a narrower and more constrained profile, suggesting that its larger architecture limits deeper membrane penetration, while L33 displayed local energetic barriers during the membrane-approach phase, probably reflecting the higher structural cost required to reorganize its more ordered helical conformation.

In addition, all the simulated peptides showed preferential cardiolipin association over POPE and POPG. Notably, although DIM-33 formed the highest absolute number of interactions, it showed the lowest number of normalized interactions, suggesting that the linear peptides may be more prone to stabilization and subsequent association with membrane phospholipids.

These results support the idea that the different oligomeric forms are not functionally redundant but instead contribute complementary membrane-interaction behaviors that may explain their cooperative performance in peptide combinations. Nevertheless, further experimental studies will be required to fully determine the extent to which these membrane-interaction behaviors contribute to cooperative antimicrobial activity.

In this context, nanopore and membrane permeabilization methodologies may represent useful future approaches to experimentally evaluate how cooperative oligomeric formulations alter membrane conductance and pore-formation dynamics.

Several limitations should be considered. First, the Combinatorial Therapeutic Index is a predictive in vitro metric and does not replace direct cytotoxicity testing of complete peptide mixtures. Its calculation assumes non-synergistic cytotoxic contributions that can be approximated by concentration addition; however, antibacterial additivity does not necessarily imply additive toxicity in mammalian cells. Second, a biased force was implemented during molecular simulations to promote peptide insertion and enable standardized comparison across peptide architectures; therefore, the simulations should be interpreted as relative measures of membrane-interaction behavior rather than as fully spontaneous insertion pathways.

## 4. Materials and Methods

### 4.1. Peptide Synthesis and Characterization

The antimicrobial peptides were synthesized by Fmoc-based Solid Phase Peptide Synthesis (Fmoc-SPPS) using a Syro multiple peptide synthesizer (MultiSynTech, Witten, Germany). Linear and dimeric peptides were assembled utilizing TentaGel S RAM resin, which requires a deprotected Lysine on the C-terminal to build core of the dimer. On the other hand, tetrameric peptides were built over TentaGel cMAP 4-branch β-Ala resin, which allows a more standardized yield for branched peptides.

During synthesis, Fmoc groups were removed with piperidine in DMF, and amino acid couplings were carried out utilizing HBTU/DIPEA-mediated activation in DMF/NMP. The selected side-chains protecting groups were Pbf for arginine, Boc for lysine, and tBu for serine. Once synthesis was completed, the peptides were cleaved from the resin and simultaneously deprotected utilizing a solution of trifluoroacetic acid (TFA), tri-isopropylsilane and water (95:2.5:2.5 *v*/*v*/*v*). Then, the crude peptide was precipitated using diethyl ether to remove TFA and other synthesis byproducts.

Purification of the crude peptide was performed through reverse-phase high-performance liquid chromatography (RP-HPLC), utilizing 0.1% TFA in water as mobile phase A and methanol as mobile phase B. Additionally, mass spectrometric analysis using Matrix-Assisted Laser Desorption/Ionization Time-of-Flight/Time-of-Flight (Ultraflex TOF/TOF mass spectrometer (Bruker Daltonics, Bremen, Germany) was employed to validate the exact molecular weight and structural integrity of the synthesized peptide. Lastly, reverse-phase chromatography analysis was performed using a Phenomenex Jupiter C18 analytical column (300 Å pore size, 5 μm particle size, 250 × 4.6 mm; Phenomenex, Torrance, CA, USA) to assess purity and confirm the absence of synthesis byproducts.

### 4.2. Eukaryotic Cell Viability Assay

Cytotoxicity assays were performed using RAW 264.7 murine macrophage cells (ATCC^®^ TIB-71™, American Type Culture Collection, Manassas, VA, USA). Cells were seeded at a density of 5 × 10^3^ cells per well in 96-well microplates and incubated for 24 h at 37 °C in a humidified atmosphere containing 5% CO_2_ to allow for proper cell attachment and establishment of baseline metabolic activity.

Following the initial incubation period, 100 μL volumes of peptide solutions were added to the wells and maintained for 24 h under the same incubation conditions. Cell viability was assessed using the well-established MTT (3-(4,5-dimethylthiazol-2-yl)-2,5-diphenyltetrazolium bromide) colorimetric assay, which measures mitochondrial metabolic activity as an indicator of cell viability. Following peptide exposure, 20 μL of MTT solution (5 mg/mL in phosphate-buffered saline) was added to each well, and plates were incubated at 37 °C for 3 h. During this incubation period, metabolically active cells reduce the yellow MTT substrate to purple formazan crystals, which accumulate within the cells.

To quantify the formazan production, 120 μL of 4 mM HCl in isopropanol was added to each well to dissolve the formazan crystals and lyse the cells, creating a homogeneous colored solution suitable for spectrophotometric analysis. Optical density measurements were performed using a microplate reader at 570 nm, with the absorbance being directly proportional to the number of viable cells. Cell viability percentages were calculated by comparing the absorbance values of peptide-treated cells with those of untreated control cells, allowing for the determination of dose-dependent cytotoxic effects. Cytotoxicity assays were performed in biological triplicate. Figure 2 and Figure 3 report mean EC50 values, while Figure 3 additionally displays the corresponding standard deviation within the graphical representation. Complete raw optical density (OD) measurements for all cytotoxicity experiments are provided in the Supplementary Data.

### 4.3. Minimal Inhibitory Concentration (MIC) Determination

Minimal Inhibitory Concentration assays were systematically performed in triplicate to ensure reproducibility and clinical relevance of the results. The assays were conducted using the standardized microdilution methodology as specified by the Clinical and Laboratory Standards Institute (CLSI) guidelines, ensuring consistency with internationally accepted protocols for antimicrobial susceptibility testing [44].

Bacterial strains were initially cultured on Mueller-Hinton (MH) agar plates (Becton Dickinson, Franklin Lakes, NJ, USA) and incubated under standard conditions to obtain isolated colonies. A single, well-isolated colony from each strain was carefully selected using a sterile cotton swab and streaked into sterile cation-supplemented Mueller-Hinton broth to create a standardized bacterial suspension. The optical density of these suspensions was precisely measured using a densitometer (Densichek, bioMérieux, Marcy l’Étoile, France) and adjusted to 0.15 McFarland standard, corresponding to approximately 5 × 10^7^ CFU/mL.

Serial two-fold dilutions of the test peptides were prepared in sterile Mueller-Hinton broth in 96-well microtiter plates, with each well containing 100 μL of peptide solution. An equal volume (100 μL) of standardized bacterial suspension was then added to each well, resulting in a final bacterial inoculum of approximately 5 × 10^5^ CFU per well in a total volume of 200 μL. This inoculum density represents the standard concentration recommended by CLSI guidelines for antimicrobial susceptibility testing.

The inoculated plates were incubated at 35 °C for 18–20 h under standard atmospheric conditions, allowing sufficient time for bacterial growth while preventing over-incubation that might lead to false results. MIC determinations were evaluated through both optical density measurements and visual inspection of bacterial growth.

### 4.4. Checkerboard Assay

The checkerboard microdilution assay was systematically employed to evaluate potential synergistic, additive, or antagonistic interactions between different structural forms of SET-M33 and its analogs, specifically comparing monomeric, dimeric, and tetrameric configurations against *Escherichia coli* TG1 as a representative Gram-negative bacterial target. This comprehensive approach allows for the assessment of how different peptide architectures might interact to enhance or diminish overall antimicrobial efficacy.

Bacterial cultures were prepared in Mueller-Hinton broth (MHB) and incubated at 37 °C with appropriate agitation to ensure optimal growth conditions. The bacterial inoculum was carefully standardized to a 0.5 McFarland turbidity standard, corresponding to approximately 1.5 × 10^8^ CFU/mL, and subsequently diluted to achieve a final working concentration of 1–5 × 10^5^ CFU/mL for use in the assay.

Peptide stock solutions were prepared as described for the MIC assay according to CLSI M07 [44]. Peptide combinations were then evaluated using a checkerboard microdilution format in 96-well microplates, where one peptide variant was serially diluted across the rows and the second peptide variant was serially diluted down the columns [45].

Following bacterial inoculation, plates were incubated at 37 °C for 18–24 h under standard conditions, and individual MIC values for each peptide combination were determined through visual assessment of bacterial growth inhibition. The Fractional Inhibitory Concentration (FIC) Index was calculated using the established formula: FIC = (MIC of peptide A in combination/MIC of peptide A alone) + (MIC of peptide B in combination/MIC of peptide B alone).

The calculated FIC values were interpreted according to standard criteria: synergistic interactions were defined as FIC ≤ 0.5, indicating that the combined effect is greater than the sum of individual effects; additive effects were characterized by 0.5 < FIC ≤ 1, suggesting that the combined effect equals the sum of individual contributions; indifferent interactions were identified by 1 < FIC ≤ 4, indicating no significant interaction between the peptides; and antagonistic effects were determined by FIC > 4, suggesting that the combination produces less effect than expected based on individual activities.

Checkerboard assays were evaluated through visual inspection of bacterial growth together with optical density measurements. Growth inhibition was only considered valid when no visible bacterial pellet formation was observed and OD values remained below an empirically defined threshold corresponding to 70% above the background signal. Complete raw OD measurements for all checkerboard experiments are provided in Supplementary Data.

### 4.5. Peptide Insertion Molecular Dynamics

All-atom molecular dynamics simulations were performed to compare the membrane-interaction behavior of the linear peptides L33 (KKIRVRLSA) and L8 (KKIRVRLVA), the dimeric form DIM-33, and the tetrabranched peptide SET-M33. Simulations were performed by GROMACS, implementing the CHARMM36m force field for peptides and lipids [46,47]. A bacterial inner-membrane model was constructed as a symmetric bilayer composed of phosphatidylethanolamine (POPE), phosphatidylglycerol (POPG), and cardiolipin (CDL2) in a 70:25:5 molar ratio. The bilayer was oriented perpendicular to the *z*-axis and embedded in a periodic simulation box of 12 × 5 × 5 nm^3^.

Initial conformations of the linear peptides were generated using CHARMM-based modeling tools, whereas the dimeric and tetrameric structures were assembled using UCSF Chimera 1.19 [48]. Peptides were placed approximately 1.5 nm above the membrane surface, with the peptide center of mass initially located at approximately z = 9.5 nm, corresponding to approximately 3.5 nm from the bilayer center. Peptides were oriented with the N-terminal region towards the membrane to standardize the initial insertion geometry across systems.

Systems were solvated with TIP3P water and neutralized with Na^+^ and Cl^−^ ions. Formal peptide charges were retained in the topology to preserve the intrinsic cationic characteristic necessary for a realistic electrostatic interaction with anionic lipid headgroups. Protonation states were assigned at pH 7 prior to topology generation using OpenBabel 3.1.0 [49]. Linear peptides carried a net charge of +4. Non-standard residues were introduced to reproduce the lysine-based branching cores of DIM-33 and SET-M33: F01 was used to model the dimeric branching point, whereas F04 was used to model the tetrabranched core containing the terminal β-alanine carboxylate. These topology modifications resulted in net charges of +10 for DIM-33 and +19 for SET-M33.

Each system was subjected to steepest-descent energy minimization, followed by sequential NVT and NPT equilibration. During equilibration, positional restraints on peptide, lipid heavy atoms, and dihedral angles were progressively relaxed, while the integration time step was increased from 1 to 2 fs. Temperature was maintained at 303.15 K using the V-rescale thermostat, and pressure was regulated semi-isotropically at 1 bar using the C-rescale barostat. Long-range electrostatics were treated using the particle mesh Ewald method with a real-space cutoff of 1.2 nm. Lennard-Jones interactions were handled using a force-switching scheme between 1.0 and 1.2 nm.

After equilibration, production simulations were performed over 300 ns utilizing four independent replicas per peptide system. To promote peptide insertion and evaluate their diffusion susceptibility under standardized conditions, a constant external force of 50 kJ·mol^−1^·nm^−1^ was applied along the *z*-axis between the center of mass of the peptide and that of the membrane, oriented toward the bilayer interior. This biased-insertion protocol was used to compare relative insertion tendencies, conformational adaptation, and peptide-lipid interaction patterns among the different oligomeric forms.

Umbrella sampling simulations were subsequently performed for L33, L8, and DIM-33 to reconstruct the peptide-membrane insertion free-energy profiles [40,41,42,43,44,45,46,47,48,49,50,51,52]. Starting configurations for each umbrella window were extracted from the 300 ns biased-insertion trajectories by selecting frames whose peptide-membrane center-of-mass distance most closely matched each target window. A harmonic restraint of 1000 kJ·mol^−1^·nm^−2^ was applied along the *z*-axis between the peptide and membrane centers of mass, with windows spaced every 0.1 nm. The sampled ranges were 1.5–2.5 nm for DIM-33, 1.0-3.2 nm for L8, and 1.2–3.5 nm for L33 (11, 23, and 24 windows, respectively), corresponding to their insertion depth. Each window was equilibrated for 5 ns and then simulated for 30 ns, yielding a total of 2030 ns. Potential of mean force profiles were reconstructed using the weighted histogram analysis method implemented in gmx wham. The first 1 ns of each production window was discarded as additional equilibration, and statistical uncertainties were estimated by bootstrapping with 200 iterations.

Peptide-membrane interactions were quantified using a panel of structural and dynamical descriptors. Membrane insertion depth was calculated as the Z-distance between the centers of mass of the peptide and the membrane. Peptide orientation was estimated from the tilt angle between the backbone N-to-C vector and the membrane normal. Backbone RMSD was used to evaluate conformational rearrangement during insertion. Solvent-accessible surface area was calculated using the measure sasa module in VMD and normalized per residue to allow fair comparison between monomeric and oligomeric systems. Peptide-lipid hydrogen bonds were quantified using MDAnalysis 2.9.0, implementing a donor-acceptor distance cutoff of 3.5 Å and a donor-hydrogen-acceptor angle cutoff of 150°. Only hydrogen bonds involving POPE, POPG, or cardiolipin headgroup atoms were considered.

Lipid-specific contact patterns were quantified using a 0.45 nm distance cutoff between peptide atoms and lipid headgroup atoms. Contact counts were normalized by the total number of molecules of each lipid species in the system. Cardiolipin enrichment was then expressed relative to POPG or POPE contacts, allowing lipid preference to be compared independently of bulk membrane composition.

Trajectory quality was assessed by visual inspection in VMD, pulling-coordinate continuity, box-dimension stability, and peptide periodic-image analysis. No relevant discontinuities, peptide fragmentation, membrane tearing, or abnormal box instabilities were detected for L33, L8, or DIM-33. SET-M33 showed systematic periodic-image contacts due to its larger molecular size relative to the simulation box; therefore, its quantitative insertion analysis was treated separately from the direct L33, L8, and DIM-33 comparison. Detailed quality-control analyses are reported in Supplementary Information.

### 4.6. Calculation of the Combinatorial Therapeutic Index

To evaluate whether peptide combinations improved the predicted therapeutic profile beyond antibacterial potency alone, we calculated a Combinatorial Therapeutic Index (CTI). This metric integrates the dose reduction achieved by each peptide in an effective antimicrobial combination with its experimentally determined cytotoxicity against RAW 264.7 macrophages. To develop this index, we built on the concepts of the Biocompatibility Index and the Dose Reduction Index [53,54].

For each peptide component *i*, the dose reduction index was calculated as follows:

$$DRI=\;\frac{{MIC}_{alone}}{{MIC}_{combination}}$$

where $MIC_{i,alone}$ is the MIC of peptide i when tested individually, and $C_{i,combination}$ is the concentration of the same peptide in the effective combination identified by checkerboard analysis.

The baseline toxic burden of a peptide pool was defined as the sum of the relative cytotoxic burden associated with administering each component at its standalone effective antibacterial concentration:

$$TB\;=\;\sum_{i}\frac{{MIC}_{alone}}{{CC50}_{i}}$$

where $CC50_{i}$ is the concentration of peptide i that reduced RAW 264.7 macrophage viability by 50%. This term corresponds to the summed inverse biocompatibility index of the peptide components.

The dose-weighted toxic burden was then calculated by replacing the standalone MIC of each component with the dose used in the effective combination. Since $DRI_{i}=MIC_{i,alone}/C_{i,combination}$, this can be expressed as follows:

$$WTB\;=\;\sum_{i}\frac{{MIC}_{i}}{{DRI}_{i}\;\cdot \;{CC50}_{i}}$$

which is equivalent to the following:

$$WTB\;=\;\sum_{i}\frac{{CI}_{combination}}{{CC50}_{i}}$$

Finally, CTI was defined as follows:

$$CTI=\;\frac{TB}{WTB}$$

Thus, CTI quantifies the predicted reduction in relative cytotoxic burden achieved by redistributing the antibacterial load across peptide components with different cytotoxic profiles. A CTI value greater than 1 indicates an improvement in the predicted therapeutic profile relative to the equivalent standalone effective doses of the same components. The calculation assumes additive cytotoxic contributions among peptide components and was used as a comparative in vitro metric rather than as a substitute for direct cytotoxicity testing of the complete peptide mixtures.

A detailed illustrative CTI calculation workflow is provided in Supplementary Figure S1.

## 5. Conclusions

This study demonstrates that SET-M33-derived antimicrobial peptides can be optimized not only through sequence modification or oligomeric engineering, but also through cooperative formulation of structurally related peptide architectures. This novel strategy allows diverse balanced oligomeric analogs to cooperate by reducing functional and clinical bottlenecks through toxic burden redistribution. The proposed Combinatorial Therapeutic Index further showed that combinations with similar FIC values can differ substantially in their predicted therapeutic profile depending on how the antibacterial load is redistributed across peptides with distinct cytotoxic profiles.

Supporting all-atom molecular simulations provided mechanistic insights, suggesting that different oligomeric forms exhibit complementary membrane-interaction behaviors, with larger architectures favoring surface engagement and smaller peptides displaying greater insertion propensity.

## Figures and Tables

**Table 1:** MIC (µg/mL) values of lysine/arginine scanning analogs against Gram-negative bacteria.

Sequence	*P. aeruginosa* PAO-1	*E. coli* TG-1	*K. pneumoniae* ATCC	Net Charge
KKIRVRLSA	1.50	1.50	0.75	4
RKIRVRLSA	1.50	1.50	6.00	4
KKRRVRLSA	50.00	>50	N/A	5
KKIRKRLSA	50.00	>50	N/A	5
KKIRRRLSA	50.00	>50	N/A	5
KKIRVRKSA	50.00	>50	N/A	5
KKIRVRRSA	50.00	>50	N/A	5
KKIRVRLKA	3.00	6.00	6.00	5
KKIRVRLRA	3.00	3.00	6.00	5
KKIRVRLSK	0.70	3.00	50.00	5
KKIRVRLSR	25.00	12.00	N/A	5

N/A, not assayed; MIC values were not determined for the indicated peptide-bacterial strain combination.

**Table 2:** MIC (µg/mL) values against targeted bacteria, including physicochemical determinants.

Sequence	*P. aeruginosa* PAO-1	*E. coli* TG-1	*K. pneumoniae* ATCC	Aliphatic Index	Boman Index	Net Charge
KKIRVRLSA	1.50	1.50	0.75	130.00	0.62	4
KKIRVRLVA	12.00	6.00	6.00	162.22	0.69	4
KKIRVRLKA	3.00	6.00	6.00	130.00	0.66	5
KKIRVRLRA	1.50	3.00	6.00	130.00	0.69	5
KKIRVRLKLK	6.00	1.50	3.00	146.00	0.72	6
KKIRVRLLA	12.00	12.00	12.00	173.33	0.71	4
KKIRVRLSI	50.00	50.00	25.00	162.22	0.70	4

**Table 3:** Oligomeric variants MIC (µg/mL) values against *E. coli* TG1.

Peptide	Oligomeric State	Sequence	MIC (µg/mL)
L33	Linear	KKIRVRLSA	3.00
L8	Linear	KKIRVRLVA	3.00
DIM-33	Dimer	KKIRVRLSA	0.75
SET-M33	Tetramer	KKIRVRLSA	0.35
SET-M8	Tetramer	KKIRVRLVA	3.00

## Data Availability

Molecular dynamics simulation data generated in this study are publicly available through the Zenodo repository: https://zenodo.org/records/20179148 (accessed on 14 May 2026). Additional experimental data supporting the findings of this study are included within the article and its Supplementary Materials or are available from the corresponding author upon reasonable request.

## Supplementary Materials

The following supporting information can be downloaded at https://www.mdpi.com/article/10.3390/antibiotics15060591/s1. Figure S1: Checkerboard/FIC analysis and CTI calculation of SET-M33-based peptide combinations; Figure S2: Cytotoxicity analysis of SET-M33 oligomeric variants and tetrameric analogs against RAW 264.7 macrophages; Figure S3: Molecular dynamics quality-control analyses and SET-M33 biased-insertion descriptors. Supplementary Data File S1: MIC (Table S1), FIC (Table S2), cytotoxicity (Table S3), CTI calculation, and molecular dynamics summary data (Table S4).

## Abbreviations

The following abbreviations are used in this manuscript:

AMP	Antimicrobial peptide
AMR	Antimicrobial resistance
CC50	50% cytotoxic concentration
CTI	Combinatorial therapeutic index
DIM-33	Dimeric form of L33
FIC	Fractional inhibitory concentration
L33	Linear monomeric form of SET-M33
L8	Linear hydrophobic analog of L33
LPS	Lipopolysaccharide
MD	Molecular dynamic
MIC	Minimum inhibitory concentration
MTT	3-(4,5-dimethylthiazol-2-yl)-2,5-diphenyltetrazolium bromide
PMF	Potential of mean force
POPE	1-palmitoyl-2-oleoyl-sn-glycero-3-phosphoethanolamine
POPG	1-palmitoyl-2-oleoyl-sn-glycero-3-phosphoglycerol
CDL2	Cardiolipin
RMSD	Root mean square deviation
SASA	Solvent-accessible surface area
WHAM	Weighted histogram analysis method
