# Supplementary material for: Cooperative Oligomeric Peptide Combinations Enhance the Predicted Therapeutic Profile of SET-M33
Source: Antibiotics (Basel). 2026 Jun 9;15(6):591. doi: 10.3390/antibiotics15060591 (PMC13295434; doi:10.3390/antibiotics15060591)

SUPPLEMENTARY MATERIAL

SUPPLEMENTARY FIGURE S1. CTI CALCULATION

Combinatorial Therapeutic Index calculation of the best FIC combinations according to Material and Methods protocol.

Entry	Combination	Ratio	FIC	DRI SET-M33	DRI DIM-33	DRI L33	DRI L8	Reference toxic burden	Combination toxic burden	CTI
1	SET-M33:DIM-33	1:1	0.75	4.2	2	—	—	0.18	0.05	3.3
2	SET-M33:DIM-33	8:1	0.62	2	9.4	—	—	0.18	0.07	2.5
3	SET-M33:L33	1:8	0.75	4.2	—	2	—	0.14	0.03	4.1
4	SET-M33:L33	2:1	0.56	2	—	75	—	0.14	0.07	2.1
5	SET-M33:DIM-33:L8	1:8:32	0.56	18.8	4	—	4	0.19	0.02	8.9
6	SET-M33:DIM-33:L8	8:1:4	0.56	2	27.5	—	32.4	0.19	0.07	2.8

SUPPLEMENTARY FIGURE S2. CYTOTOXICITY

Cytotoxicity of tetrameric analogs of SET-M33 and their oligomeric analogs, measured against RAW 264.7 murine macrophage cells.

Entry	Peptide / Analog	Sequence	Oligomeric state	Aliphatic index	EC50/CC50 (μM)	Relative toxicity vs. SET-M33	Dataset
1	SET-M33	KKIRVRLSA	Tetramer	130	5.7	1	Oligomeric forms
2	DIM-33	KKIRVRLSA	Dimer	130	33.5	0.17	Oligomeric forms
3	L8	KKIRVRLVA	Linear	162	508	0.01	Oligomeric forms
4	L33	KKIRVRLSA	Linear	130	801	0.007	Oligomeric forms
5	Analog 1	KKRVRLSA	Tetramer	97	20	0.4	SET-M33 analogs
6	Analog 2	KKIRVRLSK	Tetramer	119	14	0.57	SET-M33 analogs
7	Analog 3 / SET-M33	KKIRVRLSA	Tetramer	130	8	1	SET-M33 analogs
8	Analog 4	KKIRVRLKLK	Tetramer	146	5.5	1.45	SET-M33 analogs
9	Analog 5	KKIRVRLSI	Tetramer	162	3	2.67	SET-M33 analogs

SUPPLEMENTARY FIGURE S3. MOLECULAR DYNAMICS

Box-dimension stability of the linear and dimeric systems, indicating well-equilibrated anisotropic simulation boxes suitable for peptide-insertion analyses.

System	Replica	Initial box X/Y/Z (nm)	Final box X/Y/Z (nm)	XY drift (nm)	Z drift (nm)
DIM-33	rep3	5.038 / 5.038 / 11.361	4.974 / 4.974 / 11.685	-0.064	0.324
DIM-33	rep4	5.038 / 5.038 / 11.361	4.839 / 4.839 / 12.263	-0.199	0.902
DIM-33	rep5	5.038 / 5.038 / 11.361	5.089 / 5.089 / 11.118	0.051	-0.243
DIM-33	rep6	5.038 / 5.038 / 11.361	5.075 / 5.075 / 11.211	0.037	-0.150
**DIM-33**	**Mean ± SD**	**—**	**—**	**-0.044 ± 0.100**	**0.208 ± 0.455**
L8	rep1	4.912 / 4.912 / 11.804	5.016 / 5.016 / 11.334	0.103	-0.470
L8	rep2	4.912 / 4.912 / 11.804	5.148 / 5.148 / 10.848	0.236	-0.956
L8	rep3	4.965 / 4.965 / 11.637	5.124 / 5.124 / 10.952	0.159	-0.684
L8	rep4	4.965 / 4.965 / 11.637	5.016 / 5.016 / 11.335	0.052	-0.301
**L8**	**Mean ± SD**	**—**	**—**	**0.137 ± 0.068**	**-0.603 ± 0.245**
L33	rep1	4.953 / 4.953 / 11.686	5.057 / 5.057 / 11.198	0.104	-0.489
L33	rep2	4.953 / 4.953 / 11.686	4.930 / 4.930 / 11.797	-0.023	0.111
L33	rep3	4.994 / 4.994 / 11.443	5.037 / 5.037 / 11.350	0.043	-0.093
L33	rep4	4.994 / 4.994 / 11.443	5.104 / 5.104 / 11.003	0.110	-0.440
**L33**	**Mean ± SD**	**—**	**—**	**0.058 ± 0.054**	**-0.228 ± 0.248**

Simulation-jump analysis of the linear and dimeric systems, exhibiting no significant discontinuities during peptide-insertion simulations.

System	Maximum jump per replica (nm)	Overall maximum jump (nm)	Mean maximum jump (nm)
DIM-33	0.0152, 0.0141, 0.0146, 0.0144	0.015	0.015
L8	0.0224, 0.0218, 0.0222, 0.0208	0.022	0.022
L33	0.0218, 0.0201, 0.0207, 0.0201	0.022	0.021

Initial-frame distribution of the linear and dimeric systems selected for umbrella sampling.

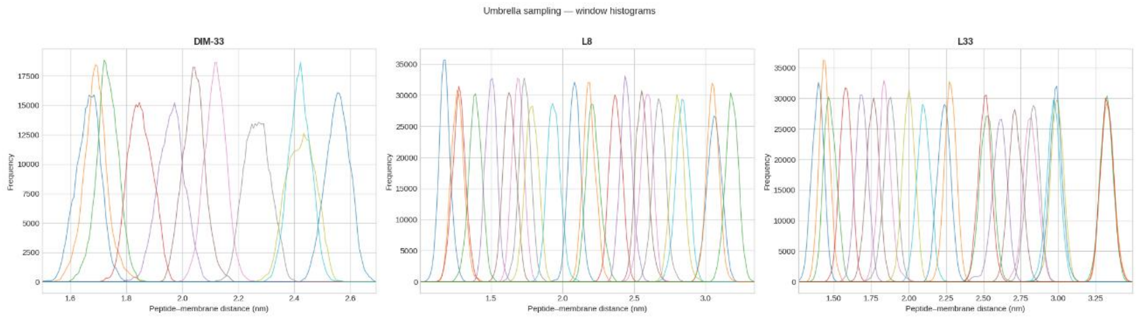

Molecular dynamics determinants measured over 300 ns simulations for SET-M33, which was excluded from quantitative analysis due to PBC-related artifacts. The tetramer exhibited the most superficial biased insertion and a more stable RMSD profile, supporting the idea that larger peptides are more constrained by steric effects and reduced flexibility, which makes diffusion into the membrane more difficult.

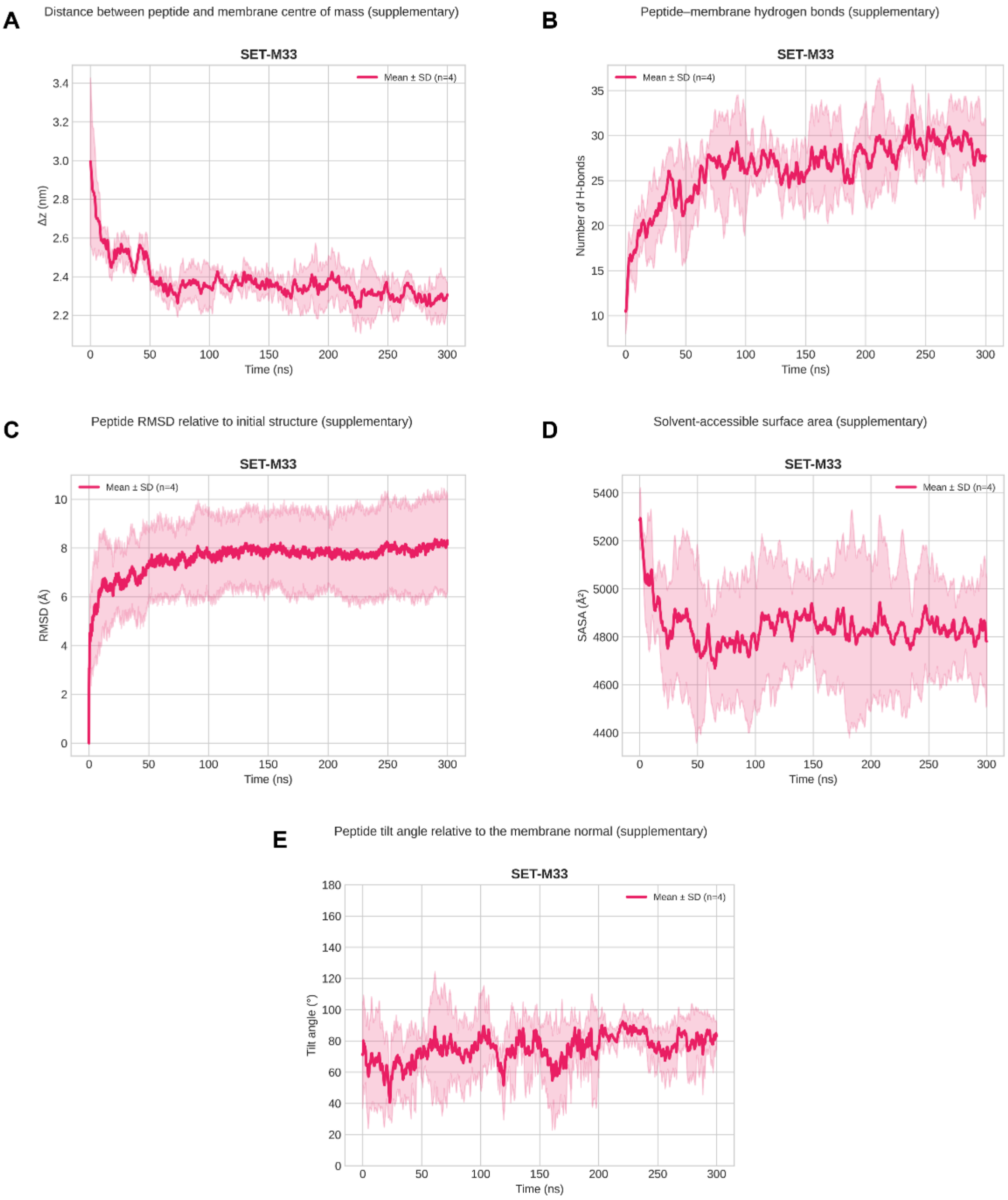

Supplement: Supplementary file 1 [file antibiotics-15-00591-s001.zip › supplementary data - cooperative.pdf]
